# Fairly flexible: brown-tufted capuchins and a squirrel monkey adjust their motor responses in a foraging task

**DOI:** 10.7717/peerj.19023

**Published:** 2025-03-12

**Authors:** Renee C. Russell, Colleen M. Buckley, Carly B. Rovner, Peter G. Judge

**Affiliations:** 1 Animal Behavior Program, Bucknell University, Lewisburg, Pennsylvania, United States; 2 Biology Department, Bucknell University, Lewisburg, Pennsylvania, United States; 3 Psychology Department, Bucknell University, Lewisburg, Pennsylvania, United States

**Keywords:** Flexibility, Habit formation, Causal understanding, Conservatism, Capuchin, Squirrel monkey

## Abstract

Prior research on non-human primates has produced contradictory results regarding behavioral flexibility and habit formation. Most observational studies of wild primates show flexibility in foraging behavior, whereas experimental data suggest captive primates tend to form habits, thus displaying conservative tendencies. Jacobson and Hopper (2019) proposed and supported the hypothesis that captive apes’ conservatism resulted from causally-unclear experimental apparatuses rather than a lack of flexibility as previous studies concluded. We replicated the experiment conducted by Jacobson and Hopper (2019) on apes with 18 brown capuchin monkeys (*Cebus* [*Sapajus*] *apella*) and five squirrel monkeys (*Saimiri sciureus*). Our goal was to investigate if they showed a similar degree of flexibility to chimpanzees (*Pan troglodytes*) and western lowland gorillas (*Gorilla gorilla gorilla*) when presented with a causally-clear task. Thus, the primary aim of this study was to determine whether this task was causally clear to monkeys, and if so, to compare their performance to that of apes. Monkeys were presented with a baited, clear tube where the removal of rods would allow the reward to drop, thus enabling the subject to retrieve said reward. Phase 1 of the study allowed us to determine whether the monkeys had a causal understanding of the task and provided an opportunity for habits to develop. Phase 2 presented the monkeys with a new reward configuration, requiring the removal of fewer rods to retrieve the reward to test if their causal understanding of the task would result in a flexible, more efficient response. The capuchins demonstrated cognitive flexibility and possible causal understanding in a manner similar to that of the apes. However, only one of five squirrel monkeys was efficient, suggesting the majority may not have understood a causal relationship between removing the rods and receiving the reward. Our study supports Jacobson and Hopper’s (2019) conclusion that causally-clear tasks reduce habit formation and conservatism in capuchins, but more evidence is needed with respect to squirrel monkeys.

## Introduction

Many primate species show some degree of behavioral flexibility. In the context of this study, we define flexibility as an intentional variation in behavior based on the demands of the task. This flexibility is an important aspect of feeding and foraging behavior, effectively reducing competition over limited food resources in the wild (Chapman, 1987). Flexibility is advantageous, as it allows for more efficient utilization of existing resources or exploitation of new resources (Huebner & Fichtel, 2015). Flexibility in primates has been studied both observationally and experimentally, showing that primates can be flexible in how and where they forage. Primates in the wild have been observed to display flexibility in how they eat, with different species using different tools to access resources, or even diverse cultures within the same species employing variegated tool use (chimpanzees, *Pan troglodytes*: Luncz & Boesch, 2014; bearded capuchins, *Sapajus libidinosus*; Luncz et al., 2016).

Flexibility has been thoroughly explored in chimpanzees. Experimentally, they have been shown to flexibly adjust foraging locations and techniques in order to maximize the reward they receive (Hopper et al., 2015; Van Leeuwen et al., 2013). Wild chimpanzees have also displayed flexibility in their behavioral repertoire. Luncz & Boesch (2014) observed neighboring chimpanzee communities creating different tools despite similar food types and material availability. Sakura & Matsuzawa (1991) also found that wild chimpanzees utilize varied materials as tools depending on availability. These chimpanzees even tried using the same stone as two distinct tools, a hammer and an anvil. They flexibly adjusted the stone’s use to determine the way in which it optimally functioned. Western gorillas (*Gorilla gorilla*) have also been shown to display some behavioral flexibility. Manrique, Völter & Call (2013) showed that gorillas, amongst other great apes, were able to abandon known problem-solving strategies and create new ones as the task evolved. Other studies have shown that flexibility is not limited to apes.

Luncz et al. (2016) observed wild bearded capuchins utilizing different tools depending on the type of nut available. Another study on wild bearded capuchins showed their ability to incorporate novel food sources, including human foods, into their diet (Sabbatini et al., 2008). Sabbatini et al. (2008) observed capuchins adjusting their feeding strategies, locations, and diet in order to maximize resource acquisition. Flexibility in tufted capuchins (*Sapajus* spp.) has also been shown experimentally, where they modified their choices when presented with different scenarios (De Petrillo et al., 2015). De Petrillo et al. (2015) presented capuchins with a series of choices between “safe” and “risky” options. The safe option guaranteed four food items, while the risky option was either one or seven food items. The risky option was presented in three conditions: “neutral” (50% chance of receiving one item, 50% chance of receiving seven items), “advantageous” (33% chance of receiving one item, 67% chance of receiving seven items), and “disadvantageous” (67% chance of receiving one item, 33% chance of receiving seven items). The capuchins avoided the neutral choice when presented with the advantageous-neutral pair, but chose the neutral option when presented with the neutral-disadvantageous pair. In summary, the literature pool suggests that capuchins alter their foraging behavior and tool use to their advantage. This indicates that capuchins may possess the ability to display flexible behavior in a similar capacity to that of chimpanzees.

Flexibility has been examined less thoroughly in squirrel monkeys (*Saimiri sciureus*). Anderson et al. (2010) revealed that captive squirrel monkeys altered their gestural communication in response to eye contact from an experimenter. The monkeys made longer and more frequent gestural requests for food under two conditions: when the food was visibly available and when the experimenter was looking directly at the monkey. Additionally, the squirrel monkeys used in the present study flexibly changed their choices to obtain the highest-valued reward available in a token exchange task (R Russell, C Early, M Painter, P Judge, 2018, unpublished data).

Several other experiments allow us to further comment on the flexibility of squirrel monkeys despite it not being a primary objective of the studies. McKenzie et al. (2004) and Naqshbandi & Roberts (2006) investigated how squirrel monkeys anticipate future events. Squirrel monkeys demonstrated flexible behavior by adjusting their initial baseline preferences when presented with experimental contingencies. McKenzie et al. (2004) conducted several experiments where squirrel monkeys were provided with a choice between larger and smaller amounts of peanuts. In the baseline controls, they found that both subjects significantly preferred the larger amount. However, in their experimental phases, they altered the paradigm by either punishing subjects who chose the larger quantity or by rewarding the subjects who chose the smaller quantity. Subjects demonstrated behavioral flexibility by adjusting their initial baseline preference in response to the imposed consequences for their choices. For example, in Experiment 6, if the subject chose the larger number of peanuts (20) the experimenter returned after 15 min and took any uneaten nuts. Since the subjects could only eat approximately six to eight peanuts in the 15-min time period, it made the initial, smaller choice of 10 peanuts more advantageous because no peanuts were removed by the experimenter. Furthermore, in Experiment 7, if the subject chose the smaller number of peanuts—two as opposed to four—the experimenter returned 15 min later to give the squirrel monkey eight additional peanuts, bringing their total reward to 10 peanuts. In both experiments presented above, subjects demonstrated behavioral flexibility by adjusting their initial baseline preference of the initial larger amount to favor the option that ultimately provided them with the most peanuts. Naqshbandi & Roberts (2006) corroborated their findings by demonstrating that squirrel monkeys learned to choose a smaller reward over a larger one in order to have their water bottle returned to them sooner, effectively showing that squirrel monkeys could alter their baseline preferences to achieve a desired outcome.

Another important concept related to behavioral flexibility is habit formation. Flexible behavior implies that the individual is assessing all options and choosing one that is adaptive or advantageous (Brown & Tait, 2010). Habit formation, on the other hand, is utilized when decision making becomes too slow and cognitively demanding. In humans (*Homo sapiens*), a stimulus from a prior task may influence the individual to default to the previously successful response, regardless of its relative efficiency (Dezfouli & Balleine, 2013). For example, if a person learns that pushing a lever down produces a reward, they will likely continue to push the lever down upon seeing it, even if a different sequence of actions would produce a greater reward. The simple presence of the lever elicits the habitual response, even if presented with other options.

Both Hanus & Call (2011) as well as Jacobson & Hopper (2019) explored behavioral flexibility and habit formation in regards to a causally-clear task in chimpanzees and western lowland gorillas (*Gorilla gorilla gorilla*). Hanus & Call (2011) found that chimpanzees have greater success with problem-solving tasks that provided them with causally relevant clues. Based on these findings, Jacobson & Hopper (2019) hypothesized that the use of causally-unclear tasks resulted in inconsistent results across studies due to misleading evidence in favor of behaviorally conservative tendencies. Jacobson & Hopper (2019) theorized that apes may be better able to exhibit flexible behavior and adopt more efficient strategies when the causal mechanisms of the task were clear to them. They also predicted that habit formation might be indicative of a lack of causal understanding since the stimulus would elicit the action as opposed to the consequences of eliciting the action. While we cannot eliminate stimulus enhancement as a possibility, we do not consider these two mental processes as mutually exclusive and suggest that both could be occurring simultaneously. However, the fact that the apes’ first response was not exclusively to pull the rod on which the reward was placed (rod 4 first in phase 1), does not support the hypothesis that stimulus enhancement was at play here.

The apparatus presented to the apes was a clear cylindrical plastic tube with holes drilled linearly to allow rods to slide through the tube across a horizontal plane. A food reward could be held up by any one of the rods within the subject’s view. When the rods directly below the reward were removed, the reward would fall through the tube and within the subject’s reach. The location of the reward changed for phase 2, such that subjects could remove fewer rods to release the reward. The transparency of the apparatus allowed for immediate visual feedback as the reward fell, and took advantage of apes’ understanding of gravity (Tomonaga et al., 2007). The study differed from previous work because the apes did not need to change the type of action they performed, just the number of actions required to succeed (Lehner, Burkart & van Schaik, 2011; Manrique, Völter & Call, 2013).

Jacobson & Hopper’s (2019) study involved two phases. In the first phase, the apparatus was set up such that four rods were below the reward, and one above it. This phase tested subjects until they were deemed “consistently efficient,” defined as removing the four rods below the reward, and not the rod above it. The removal of any rod above the reward was considered inefficient, as it indicated an inflexible, and therefore habitual, response. Subjects had to achieve 80% efficiency on at least 20 trials to move onto the second phase. In phase 2, subjects completed 20 trials where two rods were placed below the reward, and three above it. The new, more efficient solution was to remove only the two rods below the reward. Although there was individual variation in the pattern of rods removed, none of the apes developed an exclusive pattern and all apes were able to switch to a more efficient rod-removal sequence when the configuration of the task changed.

In the current study, we adopted the apparatus design used by Jacobson & Hopper (2019) and scaled down the proportions for use in brown capuchins (*Cebus* [*Sapajus*] *apella*) and squirrel monkeys. It should be noted that this apparatus was also adapted for use in young children by Hopper, Jacobson & Howard (2020); however, we constructed our apparatus before this 2020 article had been published, and so it did not inspire our design. In an effort to produce results that could be used for meaningful cross-species comparisons, we also adopted Jacobson & Hopper’s (2019) language (such as ‘efficient’ and ‘habitual’), as well as their same procedure.

We did not expect the capuchins to execute the same level of success as chimpanzees due to their comparatively worse understanding of spatial relationships (Fujita, Kuroshima & Asai, 2003). In previous studies that compared chimpanzee and capuchin understanding of causal relationships, chimpanzees displayed consistent understanding while capuchins demonstrated less understanding (Limongelli, Boysen & Visalberghi, 1995; Sabbatini et al., 2012; Visalberghi, Fragaszy & Savage-Rumbaugh, 1995; Visalberghi & Limongelli, 1994). Based on Gunst, Boinski & Fragaszy’s (2010) finding that capuchins successfully assessed payoff rates, we predicted the capuchins would be able to adjust their behavior to obtain the reward efficiently, thus conserving energy. Bearded capuchins have been observed to adapt foraging behavior to specifically select tools based on food properties in order to optimize energy conservation (Luncz et al., 2016; Fragaszy et al., 2010). Bearded capuchins have also reportedly made responses more efficient by modifying the force at which they hit a nut depending on its state (Mangalam & Fragaszy, 2015). Given all prior evidence, we predicted that brown capuchins would minimize their effort and maximize efficiency to retrieve the reward in the puzzle task presented in this study. We thought it would be reasonable to hypothesize that capuchins might take slightly longer to “pass criterion” (*i.e*., learn the contingencies of the task in phase 1) than the apes, but then be able to apply their knowledge to phase 2 just as seamlessly as the apes.

We expected that squirrel monkeys would use more inefficient methods and have less causal understanding of the task compared to the capuchins due to past observations of their relatively short attention spans and high distractibility (Fragaszy, 1985). We were encouraged, however, by the fact that squirrel monkeys have demonstrated the ability to flexibly use learned information in an exchange task to optimize their rewards (R Russell, C Early, M Painter, P Judge, 2018, unpublished data). Additionally, their display of cognitive abilities in a series of prior self-control tasks (Russell, 2019) made the squirrel monkeys utilized in this study promising candidates for success. McKenzie et al. (2004) and Naqshbandi & Roberts (2006) showed that squirrel monkeys were able to change their initial preferences in order to achieve a desired outcome in the future. Based on this evidence, we predicted the squirrel monkeys would have the cognitive capacity to use efficient methods to retrieve the reward.

## Materials and Methods

### Subjects and housing

All monkeys that participated in this study were socially housed at Bucknell University’s Animal Behavior Laboratory in Lewisburg, Pennsylvania, United States. Twelve female and six male capuchins were tested for a total of 18 capuchin participants ranging in age from four to 24 (Table 1). The colony was established in 2000 from six monkeys acquired from Yerkes National Primate Research Center in Atlanta, GA. The capuchins were housed in an indoor enclosure complete with plastic paneled walls, linoleum floors covered with wood chip bedding, steel wire caging, and assorted metal and plastic shelves and perches. The enclosure was divided into 17 interconnected compartments with dimensions that averaged 2 m × 1.8 m × 2.4 m. Each test session occurred in the same compartment with the test subject physically isolated from the other monkeys by closing interconnecting doorways. When the monkeys were not participating in the experiment, they were free to roam all other compartments of the enclosure.

**Table 1:** Monkey participants’ identification, species, age and sex.

Individual	Species	Age	Sex
Ec	*Saimiri sciureus*	14	F
Gw	*Saimiri sciureus*	14	F
Ar	*Saimiri sciureus*	12	F
Co	*Saimiri sciureus*	12	F
Vt	*Saimiri sciureus*	12	F
Mt	*Cebus [Sapajus] apella*	24	M
Dv	*Cebus [Sapajus] apella*	24	M
Nt	*Cebus [Sapajus] apella*	24	F
De	*Cebus [Sapajus] apella*	18	F
Nk	*Cebus [Sapajus] apella*	17	F
Nw	*Cebus [Sapajus] apella*	16	F
Sd	*Cebus [Sapajus] apella*	13	F
Nb	*Cebus [Sapajus] apella*	11	F
Sg	*Cebus [Sapajus] apella*	11	F
Ny	*Cebus [Sapajus] apella*	9	M
St	*Cebus [Sapajus] apella*	9	F
Ng	*Cebus [Sapajus] apella*	7	F
Sv	*Cebus [Sapajus] apella*	6	F
Nm	*Cebus [Sapajus] apella*	6	F
Nv	*Cebus [Sapajus] apella*	4	M
Sn	*Cebus [Sapajus] apella*	4	M
Nl	*Cebus [Sapajus] apella*	4	F
Nr	*Cebus [Sapajus] apella*	4	M

Five female squirrel monkeys ranging in age 12 to 14 participated in the study (Table 1). The monkeys were acquired from Stanford University in 2012 and were added to an established squirrel monkey colony. At the time of this study, one monkey from the original colony remained in their social group, but was not tested due to age-related motor and visual impairments. The squirrel monkeys were housed in an indoor-outdoor enclosure and were tested in the indoor portion, which consisted of three interconnected compartments. Each test session occurred in the same compartment, with the test subject physically isolated from the other monkeys by closing interconnecting doorways. The squirrel monkeys were free to roam the two other compartments of the enclosure when they were not actively participating in the experiment. The total indoor portion of the enclosure measured approximately 5.3 m × 5.8 m × 2.3 m and was structurally similar to the capuchin enclosure. Both species continually had access to various objects for enrichment as well as a constant supply of water. The monkeys were fed assorted nuts, grains, fruits, and vegetables twice a day according to routine caretaking procedures.

All monkeys had previous experience participating in cognitive tests with experimenters except the two youngest capuchin monkeys (Nl and Nr). Testing included a variety of object-choice tasks, object manipulation tasks, and tool-use tasks (*e.g*., Berhane & Gazes, 2020; Essler et al., 2017; Gazes, Billas & Schmitt, 2018; Judge & Bruno, 2012; Judge & Essler, 2013; Marsh et al., 2015; Painter, Russell & Judge, 2019; Russell, 2019; Vining & Marsh, 2015; Zander & Judge, 2015; Zander, Weiss & Judge, 2013). However, none of these tasks were similar to the puzzle-like apparatus used in this study.

### Ethical note

The enclosures and husbandry procedures complied with the National Research Council’s (United States) *Guide for the Care and Use of Laboratory Animals* (Committee for the Update of the Guide for the Care and Use of Laboratory Animals, 2011). Bucknell University’s Institutional Animal Care and Use Committee approved all experimental procedures (Protocol# PGJ-19) and husbandry practices.

### Apparatus

**Figure 1:**
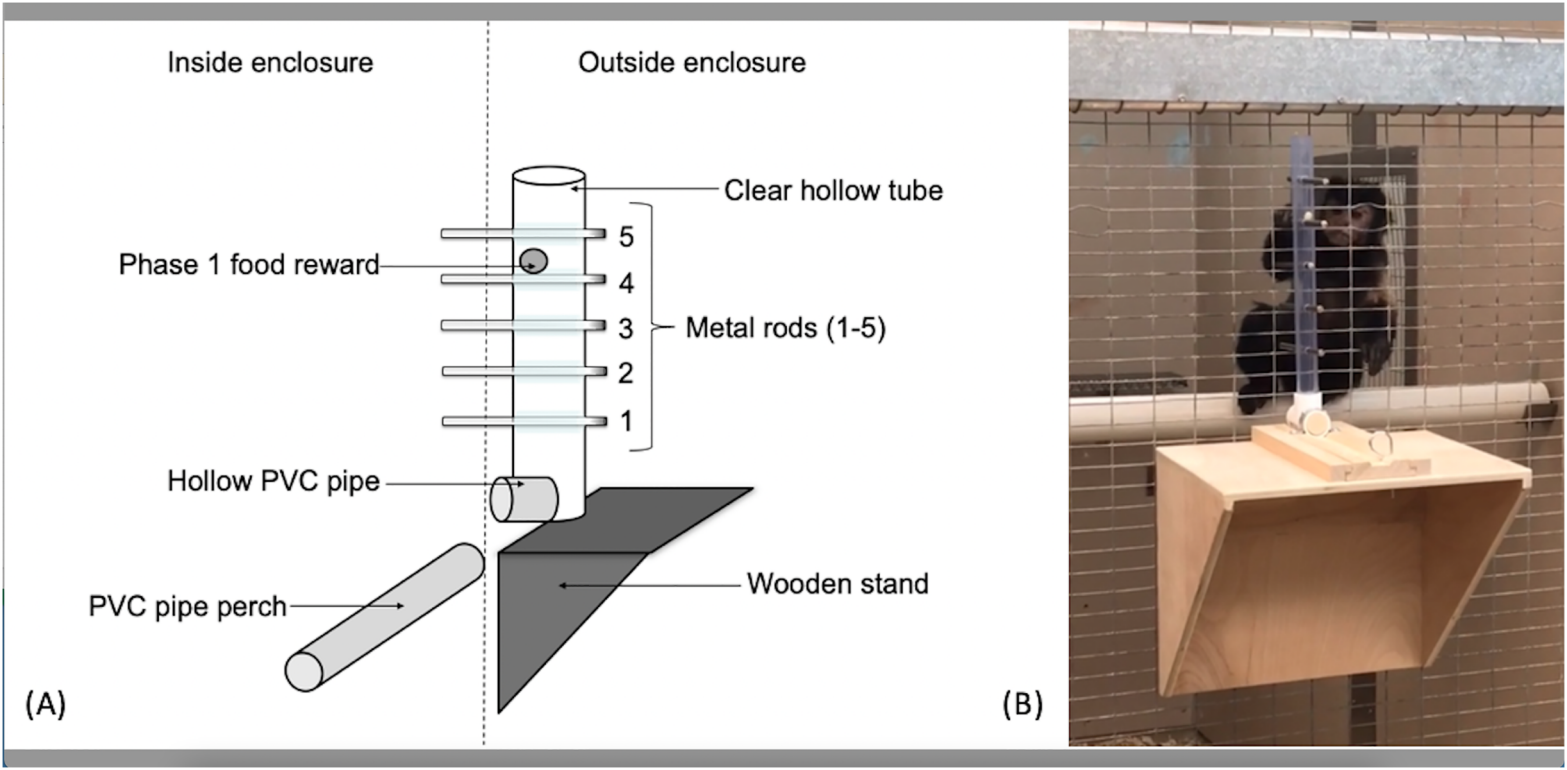
The “Monk-plunk” apparatus. (A) Diagram of the labeled apparatus. (B) A capuchin (Sd) with the apparatus conducting a phase 1 trial.

The apparatus used for the capuchins was a clear PVC tube (56 cm long, 3.2 cm in diameter) attached to the outside of the cage wire (Fig. 1). Five holes, spaced 5.08 cm apart, were drilled linearly into the tube along the same plane. The apparatus used for the squirrel monkeys utilized the same kind of PVC tube, but was 15.5 cm in length with holes drilled 2.54 cm apart to adjust for their smaller body size. Solid metal rods (15.24 cm long × 0.65 cm diameter) extended through the tube and mesh caging to become accessible to the monkeys. The PVC tube was affixed to a sliding piece of wood that allowed the experimenters to reset the apparatus outside the subjects’ reach. The sliding piece was secured with a small metal pin that fit through both the sliding piece of wood as well as the wooden base so the apparatus could not move during the trials. When the metal pin was pulled out, the apparatus was free to move toward or away from the subject.

### Procedure

Trials for capuchin and squirrel monkeys were conducted in an identical fashion, and subjects received no training prior to testing. The monkeys were baited with food items until only one subject voluntarily moved into the active testing compartment of the enclosure. To begin a trial, we inserted the metal rods from the bottom to the top, out of reach from the subject. We baited the apparatus with a high-value food reward, a piece of spherical cereal. A wooden board with two handles was used to push the metal rods through the apparatus evenly while the apparatus was sliding toward the subject. This board also prevented the monkeys from pushing rods backwards through the tube instead of pulling towards them. A trial started when all rods were pushed through the wire caging within reach of the subject. The subject could then pull out any rod above or below the reward. A rod was considered removed if it was pulled far enough to theoretically allow the reward to fall past it. Therefore, if a rod was touched but not displaced, it was not recorded as a response. A trial ended when the reward fell into the subject’s reach. We then removed any remaining rods from the apparatus, collected used rods from the enclosure, sanitized them with detergent, and dried them to eliminate the risk of creating a stimulus enhancement bias based on scent. When tested, subjects were allotted five trials, which constituted a single session. Though the experimental trials took place in the social housing facilities of the respective species, we chose not to control for bystanders or monitor their attendance to their conspecifics based on Jacobson & Hopper’s (2019) finding that social learning did not influence the results for apes. Also of note is that one or two experimenters, as well as the wooden board mentioned above, were in front of the apparatus during testing, and the subject was contained in one of the interconnecting compartments, so bystanders had restricted visual access to a subject’s performance.

In the first phase, four rods were placed below the reward, and one above it. This phase continued for a minimum of 20 trials, or until the subject was considered consistently efficient. Consistent efficiency was defined as 80% efficiency over the 10 most recent trials. Subjects who did not display any indication of becoming consistently efficient when the number of trials approached or exceeded 100 “failed” phase 1. An “efficient” trial was completed when the subject pulled only the rods beneath the reward, leaving any rods above the reward unmoved. The order in which the rods beneath the reward were removed was not considered in determining efficiency, but was recorded to allow for an analysis of habitual behavior. The number of total trials was not predetermined, as long as a minimum of 20 trials had been completed. When a subject was determined to be consistently efficient in phase 1, they started phase 2 on the next testing day. In this phase, two rods were placed below the food reward and three were placed above it. Each subject completed at least 20 trials, and in the case of two individuals up to 50 trials, regardless of strategy used.

### Data analysis

Each trial was conducted in the presence of one or two experimenters. Every action sequence was manually documented by one of the experimenters and was additionally recorded on a Sony Handycam (HDR-CX440). Action sequences were defined as the particular order in which the rods were pulled from the apparatus (*e.g*., 2,3,4,1). An action sequence that was used more than once in subsequent trials was counted and defined as a run length, meaning the shortest run length possible was two (in phase 2).

The data were analyzed using nonparametric tests due to the small sample size and outliers. Mann-Whitney *U* tests were used to assess differences between two groups (*e.g*., species). Jacobson & Hopper (2019) used Wilcoxon Rank Sum tests to compare two species and, although we wished to make direct comparisons, we used the Mann-Whitney *U* test because it is recommended for cases in which one of the two groups has a sample size smaller than ten (Jaccard & Becker, 2010). However, there would have been no difference in conclusions reported below if we had used Wilcoxon Rank Sum tests. A Kruskal-Wallis test was used to compare three groups: the monkeys and the two ape species in Jacobson & Hopper (2019). Wilcoxon signed rank tests were used to assess differences in efficiency across phases. We used an alpha level of 0.005 to evaluate statistical significance because ten tests were conducted and the more conservative value kept experimental-wise error rate at *p* = 0.05 (0.05/10 = 0.005). As in Jacobson & Hopper (2019), we used run length to evaluate the diversity of responding. A “run” was the use of the same sequence in successive trials. If median run lengths were small, then monkeys would be using a variety of sequences.

Trials were discarded under certain conditions. If the subject did not complete the task or obtained the reward from the apparatus without pulling the necessary rods to allow the reward to fall (*e.g*., obtained the reward from the top of the apparatus or shook the rods out of the apparatus) the trial was discarded. Trials that included experimenter errors (*e.g*., placing the reward between the wrong metal rods) were also omitted from the data analysis. Any instance in which a subject attempted to pull a metal rod out of the apparatus but was unsuccessful in displacing the rod on the first try was voided. Failure to remove the rod was, at times, due to apparatus-related causes. The rod would get caught in the tubing most often from an attempt to pull the rod at an angle. This included occasions when a subject attempted to pull a rod below the reward, but because the rod was not removed easily, the subject removed a rod above the reward, making the trial inefficient. The opposite also occurred, in which a subject attempted to remove a rod above the reward, but because the rod was not easily removed, the subject completed the trial efficiently by pulling all rods beneath the reward. Both of these instances were removed from the data because the trial results might not have accurately represented the subjects’ initial attempts. Based on the above protocols, 49 of 1,737 (2.82%) total trials were discarded.

## Results

### Phase 1: Causal understanding

**Figure 2:**
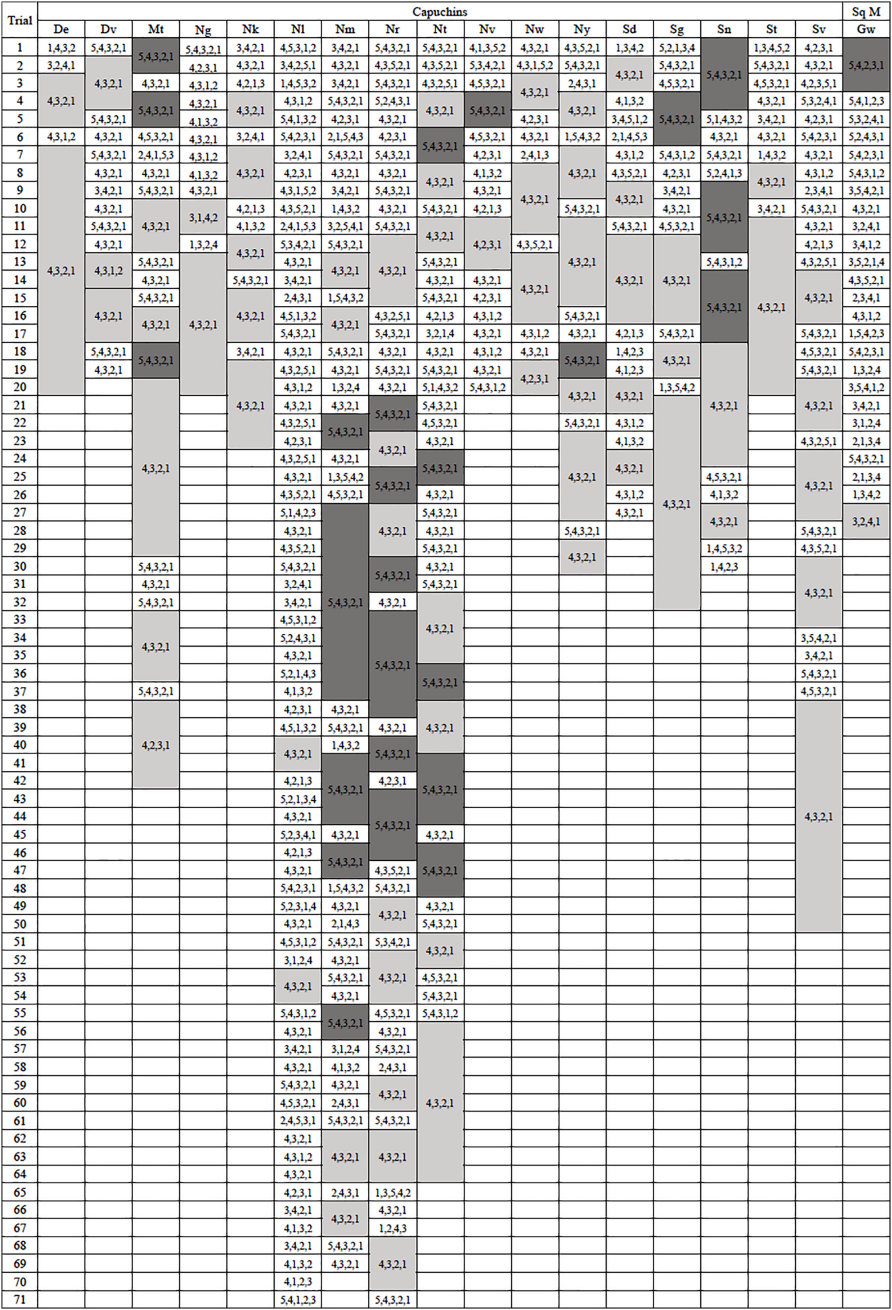
Action sequences used in phase 1. The action sequences used by each individual who passed phase 1, with efficient run lengths in light gray and inefficient run lengths in dark gray. The one capuchin and four squirrel monkeys that did not become efficient were not included in this figure. Their data can be found in the Supplemental Material.

The majority of monkeys, 21 of 23, successfully retrieved a reward the first time they were exposed to the apparatus. Of the two that did not, one chose to ignore the apparatus and did not participate while the other attempted to interact with the apparatus but did not attempt to remove any of the rods. On each of their second exposures to the apparatus, they both spontaneously utilized it without training. Only six of 23 subjects, all capuchins, efficiently extracted the reward on their first trial, exclusively removing the rods beneath the reward (Fig. 2). The capuchins were efficient for a median of 69.50% of their trials, while squirrel monkeys were efficient for a median of 13.27% of their trials. There was a significant difference between the two species (*U* = 4.0, *N* = 23, *p* = 0.002). Looking at both species, the monkeys were efficient for a median of 65.00% of their trials with one individual being efficient in every trial (De) and two individuals responding inefficiently only once before reaching criterion (Ng and Nk; Fig. 2). This result sharply contrasted with the apes’ performance in Jacobson & Hopper’s (2019) experiment where the median of efficient trials was 90.90%.

Five monkeys (one capuchin and four squirrel monkeys) never met the criterion for phase 1 after attempting the task between 94 and 122 trials. After pulling rod 5 in the majority of their last 20 trials (100%: Nb, Co, Ec, Vi; 80%: Ar), they showed no indication that they were going to exhibit causal understanding of the task (Table S1). The tendency of these monkeys to pull the top rod was evident from the start of testing. In their first 20 trials, the unsuccessful monkeys pulled the top rod in significantly more trials (mdn = 18, range 14–20) than the monkeys that became successful (mdn = 7, range 0–16; *U* = 2.5, *N* = 23, *p* < 0.001). The monkeys that failed to learn an efficient means to complete the task tended to have a top-down approach (Fig. S1). They selected the top rod first in 58.2–96.8% of their trials. They also tended to pull the rods consecutively from top to bottom, using the 5,4,3,2,1 sequence in 44.3–93.6% of their trials. As such, they developed habitual but inefficient response patterns. Since these five monkeys did not reach criterion after many trials and showed no inclination to do so, they were excluded from further data analyses and the phase 2 experiment.

Monkeys that became consistently efficient did so in a median of 29 trials. However, the most trials needed to become consistently efficient was 71. Surprisingly, the median number of trials it took for capuchins to reach the consistency criterion in phase 1 did not differ significantly from that of the apes (mdn = 21, *U* = 71.5, *N* = 31, *p* = 0.065).

### Phase 1: Action sequences

**Figure 3:**
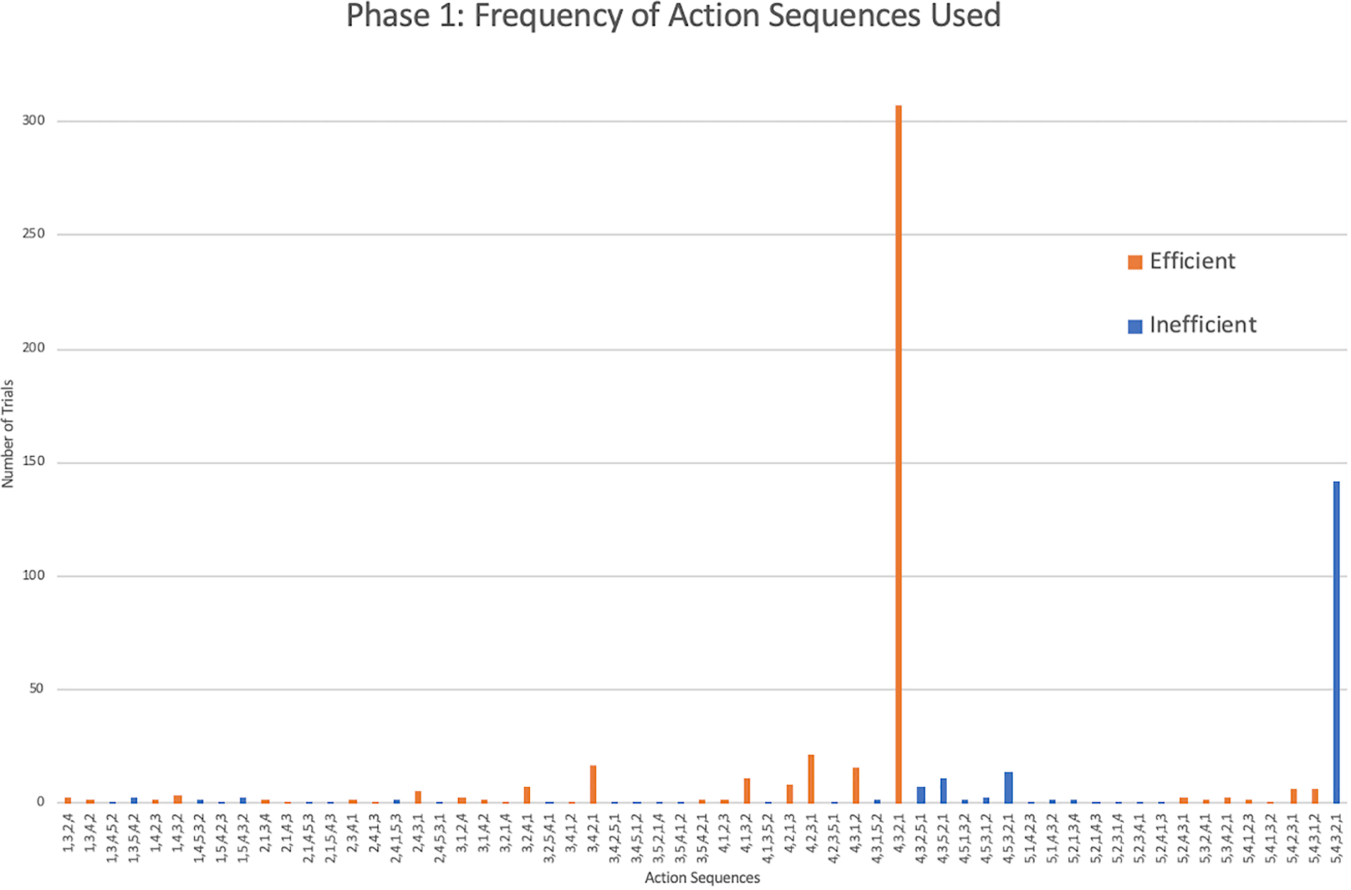
The frequency of different action sequences used in phase 1 by capuchins and the squirrel monkey that successfully passed this phase.

As seen in Jacobson & Hopper (2019), the action sequences (*i.e*., the order in which the rods were removed during a single trial) varied greatly within and between individuals. Considering only the monkeys that showed efficient responding, a total of 60 different action sequences out of the possible 120 were used during this first phase (Fig. 3). Of these 60, 22 were efficient, representing most of the possible 24 efficient sequences where rods 1 through 4 were removed in any order. The other 38 action sequences used were inefficient, representing a portion of the 96 possible inefficient action sequences where rods 1 through 5 were removed in any order. The capuchins used a total of 20 efficient and 35 inefficient action sequences. The one squirrel monkey that reached criterion used a surprisingly varied array of action sequences, utilizing a total of 21 different action sequences over 30 trials. Of those 21 action sequences, 10 were efficient.

Run lengths ranged from 2 to 14 with median run lengths per individual ranging from 2 to 8.5 (Fig. 2). The median run length for all monkeys (mdn = 3) was not significantly different from the median run lengths for the apes from Jacobson & Hopper (2019), which ranged from 2 to 5 (mdn = 2.5, *U* = 88.5, *N* = 31, *p* = 0.24).

The monkeys appeared to prefer a top-down approach (pulling rods sequentially from 4 down to 1) similar to the chimpanzees, and unlike the gorillas that tended to execute a bottom-up approach (pulling rods sequentially from 1 up to 4). Considering efficient trials from phase 1, the action sequence 4,3,2,1 was preferentially used by the monkeys (mdn = 56.45%) which was comparable to the chimpanzees’ median of 60.43%, but remarkably different from the gorillas’ median of 9.52% (Kruskal-Wallis test: *H*(2) = 12.11, *p* = 0.002). *Post hoc* tests indicated that monkeys used the 4,3,2,1 sequence significantly more than gorillas (*U* = 9.5, *N* = 25, *p* < 0.001) but were not significantly different than chimpanzees (*U* = 51.5, *N* = 24, *p* = 0.87).

### Phase 2: Flexibility

**Figure 4:**
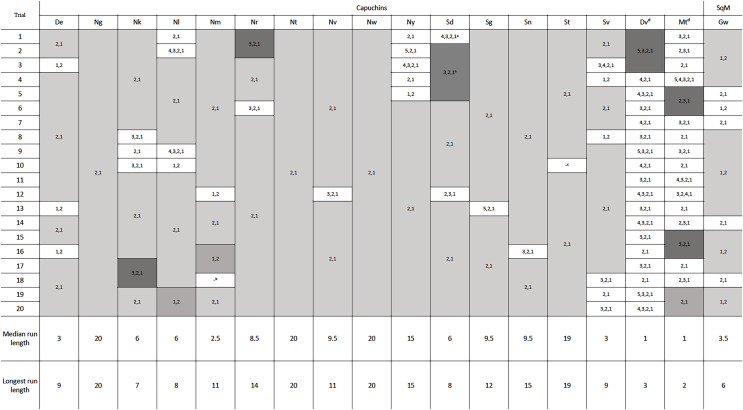
The order of rod removal by the individuals in phase 2. ‘Runs’ are shown in gray, with efficient trials in light and medium gray and inefficient in dark gray. Notes about eliminated trials: *^a^* The first five trials for Sd had the reward positioned incorrectly by the experimenters between rods 3 and 4. However, the subject completed four of these trials efficiently and was able to switch to the correct position and still perform efficiently moving forward. *^b^* One potentially efficient trial for Nm was eliminated because the subject attempted to pull rod 2 which got stuck in the apparatus. Nm then pulled out rod 1, and returned to pull out rod 2. Based on our criteria for data analysis, the trial had to be eliminated. *^c^* Outside interference compromised St’s willingness to participate in the last trial of the second session. *^d^*Dv and Mt did not pass phase 2 criteria within 50 trials. See Table S2 for their complete data.

One squirrel monkey and 17 capuchins were tested in phase 2, with the new configuration of the reward between the second and third rod. Removing four rods was specifically of interest because that was the previously efficient solution in phase 1 and the likely habitual response. Of the 18 monkeys tested, 16 adopted the efficient strategy of pulling out the bottom two rods after 20 trials (Fig. 4). Fifteen adopted the most efficient method on their first trial, only pulling rods 1 and 2. Seven individuals, including the one squirrel monkey, chose the most efficient strategy in every trial of phase 2 (Fig. 4). Six other individuals only deviated from the most efficient strategy on one or two occasions. The two monkeys that did not become consistently efficient (Dv and Mt) were both older, male capuchins. They only used the efficient strategy on 10% and 30% of their 20 trials, respectively. To determine if they might eventually learn the efficient strategy, we gave them each an additional 30 trials and they showed no indication of switching to the efficient strategy after participating in 50 total trials (Table S2). The first individual, Dv, used mostly inefficient methods right up to his 50^th^ trial. The second individual, Mt, showed some signs of adopting a more efficient strategy, but he only had 65% efficiency in the last 20 trials. As such, these two monkeys were excluded from the remaining analyses. Overall, monkeys who were consistently efficient in phase 2 were efficient for a median of 95% of individual trials. Unlike the apes in Jacobson & Hopper (2019), our monkeys that reached the efficiency criterion showed a significant improvement in their efficiency from phase 1 (mdn = 69.38%) to phase 2 (mdn = 95.00%, *Z* = 3.07, *N* = 16, *p* = 0.002). Across phases 1 and 2, chimpanzees were efficient for a median of 90.9% and 92.40% respectively, while gorillas were efficient for a median of 95.2% of trials in phase 1 and 100% of trials in phase 2, which were not significant differences.

### Phase 2: Action sequences

**Figure 5:**
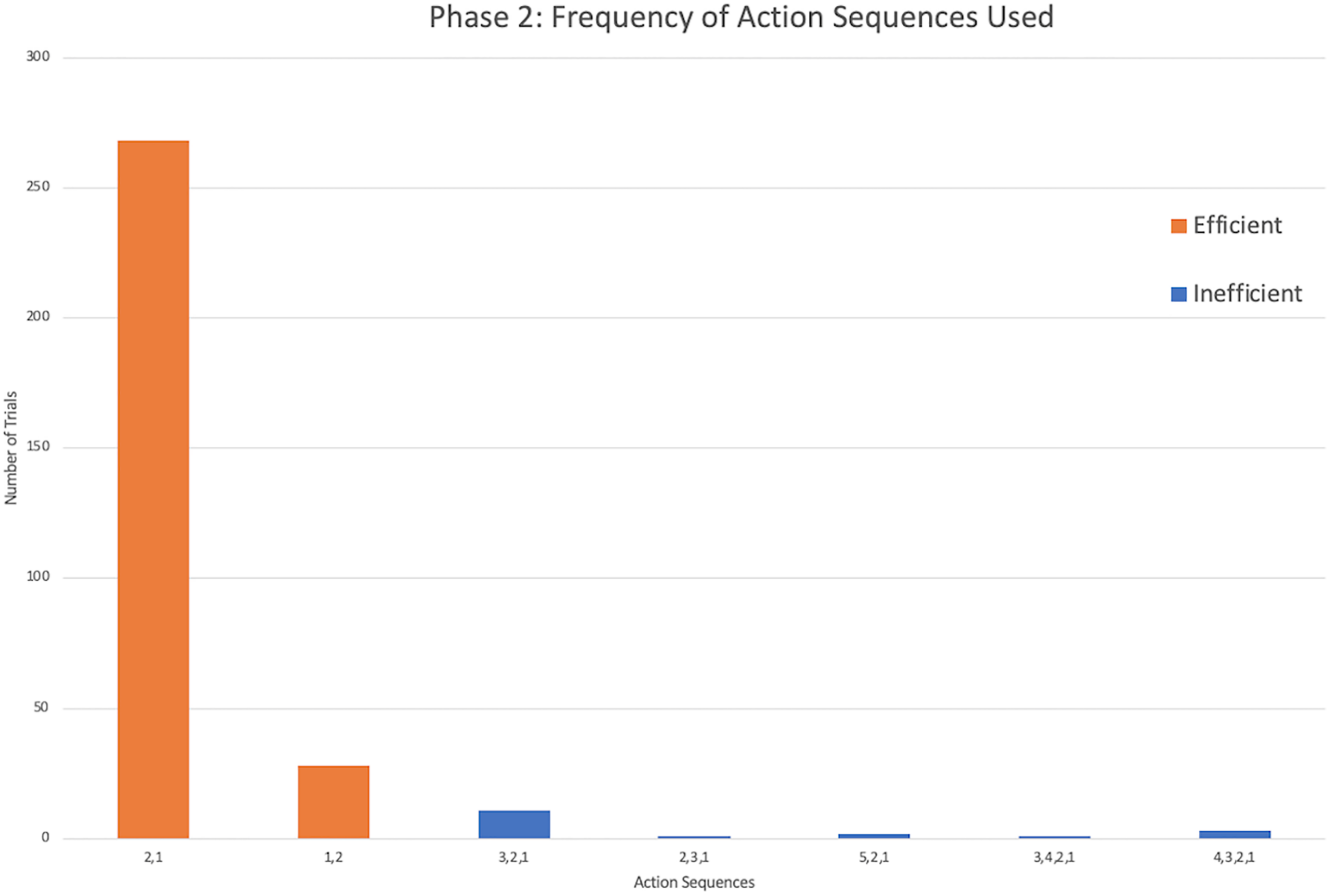
The frequency of different action sequences used in phase 2 by capuchins and the squirrel monkey that successfully passed this phase.

The successful monkeys used seven different action sequences across phase 2, including the two possible efficient strategies. Similar to phase 1, monkeys preferred the same top-down strategy as the chimpanzees, with sequence 2,1 being the majority of action sequences performed (Fig. 5). In fact, four monkeys exclusively used the 2,1 sequence across all of their trials in phase 2 (Fig. 4). Across all individuals, the monkeys used the sequence 2,1 in a median of 89.17% of trials compared to the chimpanzees’ median of 67.05% and gorillas’ 35.00%. The result indicated a significant difference between the three groups (*H*(2) = 14.55, *p* < 0.001). *Post hoc* tests indicated that the monkeys’ percentage of 2,1 sequences was not significantly higher than the chimpanzees (*U* = 16.5, *N* = 22, *p* = 0.017), but was significantly higher than the gorillas (*U* = 5.00, *N* = 23, *p* = 0.001). However, the lone squirrel monkey that met criterion (Gw) preferred the bottom-up approach, utilizing the sequence of 1,2 in 80.00% of her trials (Fig. 4). Her action sequences in phase 1 had no consistent pattern (Fig. 2).

## Discussion

To expand upon Jacobson & Hopper’s (2019) study with chimpanzees and gorillas, we tested capuchins’ and squirrel monkeys’ causal understanding and flexibility of problem-solving, effectively replicating the original experimental design. This allowed for direct comparisons between our subjects’ results and the results of the apes. Similar to Jacobson & Hopper (2019), where all apes retrieved the reward in the first trial, 21 of the 23 monkeys we tested were able to utilize the apparatus on their first attempt and retrieve the reward, regardless of their efficiency or lack thereof. The two that failed to retrieve the reward on the first attempt eventually learned how to use the apparatus without training. Therefore, all individuals displayed at least some degree of understanding of the apparatus. In Jacobson & Hopper’s (2019) apes, 11 of the 13 subjects efficiently retrieved the reward, pulling only rods beneath the reward on their first trial. Only seven of our individuals, all capuchins, efficiently utilized the apparatus in their first trial. There were ultimately 18 monkeys (17 capuchins and one squirrel monkey) that passed on to phase 2, displaying a possible causal understanding of the task. The performance differences between monkeys and apes in the initial trial support previous findings as well as our hypothesis that capuchins would not have spatial and causal reasoning equivalent to that of the apes. However, the 17 capuchins that passed phase 1 support our hypothesis that capuchins would, to some degree, show causal understanding. Only one of five squirrel monkeys passed phase 1, confirming our prediction that squirrel monkeys would not be as proficient as capuchins in this task. To summarize, our monkeys may have demonstrated a causal understanding of the task, but Jacobson & Hopper’s (2019) apes were able to grasp it more spontaneously and ubiquitously.

The monkeys that passed phase 1 showed consistent efficiency and utilized different action sequences throughout the course of the phase, further suggesting the possible causal understanding of the task at hand. It took monkeys, on average, 36 trials to become consistently efficient, while it took the apes an average of 22 trials. It is tempting to suggest that the monkeys took longer to understand the task and their goal, which would corroborate the aforementioned proposal that capuchins have a lesser understanding of spatial relationships compared to apes (Fujita, Kuroshima & Asai, 2003). However, our data analysis showed that this variation was not a statistically significant difference. These results suggest the capuchins did not acquire the task significantly slower than apes. Although this is not enough evidence to conclude that there are no species differences present, as the capuchins still required 14 additional trials to become consistently efficient. This result could be influenced by a lack of statistical power. The capuchins’ performance may be suggestive of an active learning process. While only six out of the 23 capuchins efficiently retrieved the reward in their first trial, the monkeys showed significant improvement in their efficiency from phase 1 to phase 2

The monkeys also showed a similar ability to demonstrate flexible problem-solving skills. When presented with a new configuration in phase 2, all but two of the monkeys displayed flexible behavior by removing fewer than four rods. Fourteen of the 18 monkeys tested in phase 2 used the most efficient 2-rod method in their first trial. As with the apes in Jacobson & Hopper’s (2019) study, the previous inefficient strategies were still available to the monkeys in phase 2, with no “scaffolding” transitioning to more efficient solutions. In prior work with apes, “scaffolding,” or blocking previously effective solutions, has been utilized to encourage the use of new solutions (Davis et al., 2016; Lehner, Burkart & van Schaik, 2011; Manrique, Völter & Call, 2013). Since previously effective solutions were still available and would have resulted in retrieval of the reward, this further supports the hypothesis that subjects demonstrated possible causal understanding and a flexible response. In conclusion, despite the availability of previous effective solutions, our monkeys switched to a more efficient method.

As seen with the apes in Jacobson & Hopper (2019), the majority of monkeys did not form immutable habits. In addition to the evidence provided by their flexibility transitioning from phase 1 to phase 2, they also varied the action sequences used within each phase. Our capuchins utilized 60 out of a possible 120 action sequences—22 of 24 efficient sequences, and 38 of 96 inefficient sequences. The single squirrel monkey that reached criterion used 21 sequences over 30 trials, with 10 of these being efficient. The monkeys’ tendency to try different action sequences may be explained by their possible causal understanding of the task, as suggested by Jacobson & Hopper (2019). Because they could have continued to use the first sequence that was effective, yet opted to explore new sequences and ultimately discovered most of the efficient sequences, we suggest that the monkeys, like the apes, understood the task and the apparatus.

Five monkeys did not pass phase 1, however. These monkeys never met the criterion of 80% efficiency over the 10 most recent trials, and therefore showed no indications of causally understanding the task. These monkeys appeared to form habits, tending to pull the top rod first on most trials, then continue with a top-down sequence. Interestingly, our monkeys showed significant improvement in efficiency from phase 1 to phase 2, while Jacobson & Hopper’s (2019) apes performed consistently well on both phases. This may be due to the “ceiling effect,” in which most of the apes attained the highest possible efficiency in phase 1, thus significant improvement was not possible in phase 2. A possible explanation for this is that the apes had a holistic understanding of the task mechanism from the onset of phase 1. The significant improvement in our monkeys’ efficiency could have come from an increased understanding of the mechanisms of the task in phase 2, or even simply that phase 2 required fewer actions to complete.

As with the apes, the monkeys showed substantial individual variation in the number of action sequences used and the preferred action sequences, with the longest run length (the same action sequence used in subsequent trials) in phase 1 being 14. The median run length in phase 1 did not differ significantly between the monkeys and the apes. In phase 2, all but two individuals were able to transition to being consistently efficient. The two individuals who did not transition were older, male capuchins. Interestingly, studies with humans have indicated that, as learners grow, they become more reliant on prior beliefs and less inclined to incorporate newer evidence into their behavior (Gopnik et al., 2017). This has also been observed in capuchin foraging behavior where older capuchins were observed to be less exploratory in regards to foraging strategies (Perry, 2020). This may help explain the inability for some of our older monkeys to transition to new strategies, and would make for an interesting future study utilizing this apparatus as well as more subjects within each age category.

The other 16 individuals’ results gave us a means to comment on habit formation. Jacobson & Hopper (2019) attempted to do this, but decided that their study did not provide the apes with enough trials to ascertain if habit formation occurred. Our procedure provided the opportunity for some subjects to complete more trials, allowing us to address this issue. In order to reach efficiency, some subjects completed upwards of 70 trials in phase 1 and were still able to successfully switch to the efficient method almost immediately at the start of phase 2. Based on Resende, Tavares & Tomaz’s (2003) conclusion that capuchins could form habits in as little as 15 trials, we think the monkeys had ample time to develop a habit, yet did not do so.

In Jacobson & Hopper (2019), the two species of apes preferred different action sequences. The chimpanzees typically removed top-down (4,3,2,1), while the gorillas typically removed bottom-up (1,2,3,4). The capuchins mirrored the chimpanzees, utilizing the top-down approach for a median of 56.40% of efficient trials in phase 1 and 89.10% of trials in phase 2. As with Jacobson & Hopper (2019), we feel one of the best explanations for the top-down preference is the immediate visual feedback provided by the food reward approaching the dispensing hole with each rod removed. This visual feedback serves as proximate reinforcement and perhaps acts as a form of stimulus enhancement during experimentation, which could have incentivized the capuchins and chimpanzees. Manrique, Völter & Call (2013) showed that proximate reinforcement expedited the problem-solving skills of apes, sometimes even enabling a solution, where without it, apes were unable to solve a task. This method of solving the task would also enable the capuchins to switch to the newly efficient action sequence in phase 2 by simply pulling the rods on which the food reward rested. Matthews, Paukner & Suomi (2010) revealed that capuchins were prone to stimulus enhancement and were biased to perform the first behavior that allowed them to obtain the reward. They suggested that habits could result from the interaction of stimulus enhancement and reinforcement, which may in part account for their strong bias toward the top-down method. However, this explanation does not account for the gorillas’ preference, as they should also be influenced by these factors. Although Jacobson & Hopper (2019) suggest that the top-down method could mean the subjects do not holistically understand the task, we feel that the use of this method does not preclude a holistic understanding. It is possible the monkeys had a true understanding of the task as they utilized other efficient action sequences. In phase 1, methods other than 4,3,2,1 accounted for 17.23% of trials. Additionally, in phase 2, five of the capuchins tried the bottom-up approach at least once, although they preferred using the top-down approach. Their willingness to explore other solutions could indicate that they were not using a rule-based approach to solve this task (*i.e*., pull the rod on which the reward was resting). Further, the five unsuccessful monkeys in phase 1 and the two unsuccessful monkeys in phase 2 demonstrated that there was more to understanding the task than learning this simple rule.

The sole squirrel monkey (Gw) that reached criterion utilized the largest variety of action sequences, but generally speaking, she still followed a top-down tendency in phase 1. In phase 2, she used the bottom-up method for 80% of her trials, starting with the first trial. While this individual squirrel monkey utilized efficient methods, the capuchins were still comparably more adept at the task since more individuals were able to progress to phase 2. Jacobson & Hopper (2019) raised the question of whether differences in self-control abilities could explain species differences. Previous studies with the squirrel monkeys may shed some light on this question. The five squirrel monkeys tested in this study were also tested on five different self-control tasks unrelated to this study (Russell, 2019). They all showed a surprising amount of self-control, but the individual that showed the highest level of self-control abilities performed the worst in the current study (Vi, Table S1), since she never pulled fewer than five rods. In fact, she appeared to have formed a strong habit in terms of the order in which she pulled the rods (5,4,3,2,1). Gwen (Gw), the only squirrel monkey to successfully complete the present study, performed only adequately across the self-control tasks, ranging in rank among the five monkeys from 3–5 (with 5 being the lowest). Thus, for the squirrel monkeys, self-control was not correlated with the ability to perform the present task efficiently. This leads us to suggest that the varying degrees of self-control across species may not explain interspecies differences, although a more systematic study might be warranted before any strong conclusions are drawn.

## Conclusions

We predicted that both capuchins and squirrel monkeys would demonstrate flexible behavior in phase 2 to efficiently retrieve the reward. Our predictions accounted for and expected some differences within and between our capuchins and squirrel monkeys, as well as Jacobson & Hopper (2019) apes. We found fewer species differences than we expected, however, our results did support our original hypotheses. The apes performed marginally better than the capuchins in that every single ape reached criterion in both phases, whereas not all of the capuchins did so. Additionally, the capuchins performed better than the squirrel monkeys, with only one of five squirrel monkeys able to perform the task efficiently. The performance of the apes in Jacobson & Hopper (2019) along with our capuchins and one squirrel monkey supports the conclusion that causally-clear tasks may prevent subjects from developing habit formation and conservatism that may carry energetic costs.

It would be interesting to see a future study using this apparatus to explore age and flexible behavior in order to expand upon the results of Perry (2020), since we do not believe our study population is large enough nor diverse enough to support such analyses. We also agree with Jacobson & Hopper’s (2019) suggestions for future research, namely the use of a partially opaque tube or irrelevant rods below the reward. We also suggest evaluating monkeys’ understanding of the force of gravity to determine if individuals are able to behave flexibly on a task that is not causally clear. We suggest rigging the apparatus in such a way that the reward would move up the tube against gravity when rods are removed. We believe this would obscure the causality that arises from their understanding of gravity, and would expect individuals to be more habitual and less flexible in this case. We believe Jacobson & Hopper’s (2019) apparatus is a clever and effective tool to test not only the understanding of a causally-clear task, but also behavioral conservatism *vs* flexibility. In the future, the apparatus could be modified to expand this study to other species to increase our understanding of conservative and flexible behavior across many taxa.

## Supplemental Information

10.7717/peerj.19023/supp-1 Supplemental Information 1 The frequency of different action sequences used in phase 1 by consistently inefficient monkeys.

10.7717/peerj.19023/supp-2 Supplemental Information 2 Rod pulling sequences for unsuccessful subjects in successive trials in phase 1. Grey boxes indicate successive runs of the same sequence.

10.7717/peerj.19023/supp-3 Supplemental Information 3 Rod pulling sequences for unsuccessful subjects in successive trials in phase 2. Grey boxes indicate successive runs of the same sequence.

10.7717/peerj.19023/supp-4 Supplemental Information 4 Rod pulling sequences for each successful subject in each successive trial in phases 1 and 2 with grey boxes indicating runs of the same sequence (Table 2 and Table 3). The rod pulling sequences for unsuccessful subjects in phase 1 (Table S1) and unsuccessful subjects in phase 2 (Table S2) are also available.

## Additional Information and Declarations

### Data Availability

The following information was supplied regarding data availability:

The raw data are available in the Supplemental Files.
