# Supplementary figures and images for: Fairly flexible: brown-tufted capuchins and a squirrel monkey adjust their motor responses in a foraging task
Source: PeerJ. 2025 Mar 12;13:e19023. doi: 10.7717/peerj.19023 (PMC11910149; doi:10.7717/peerj.19023)

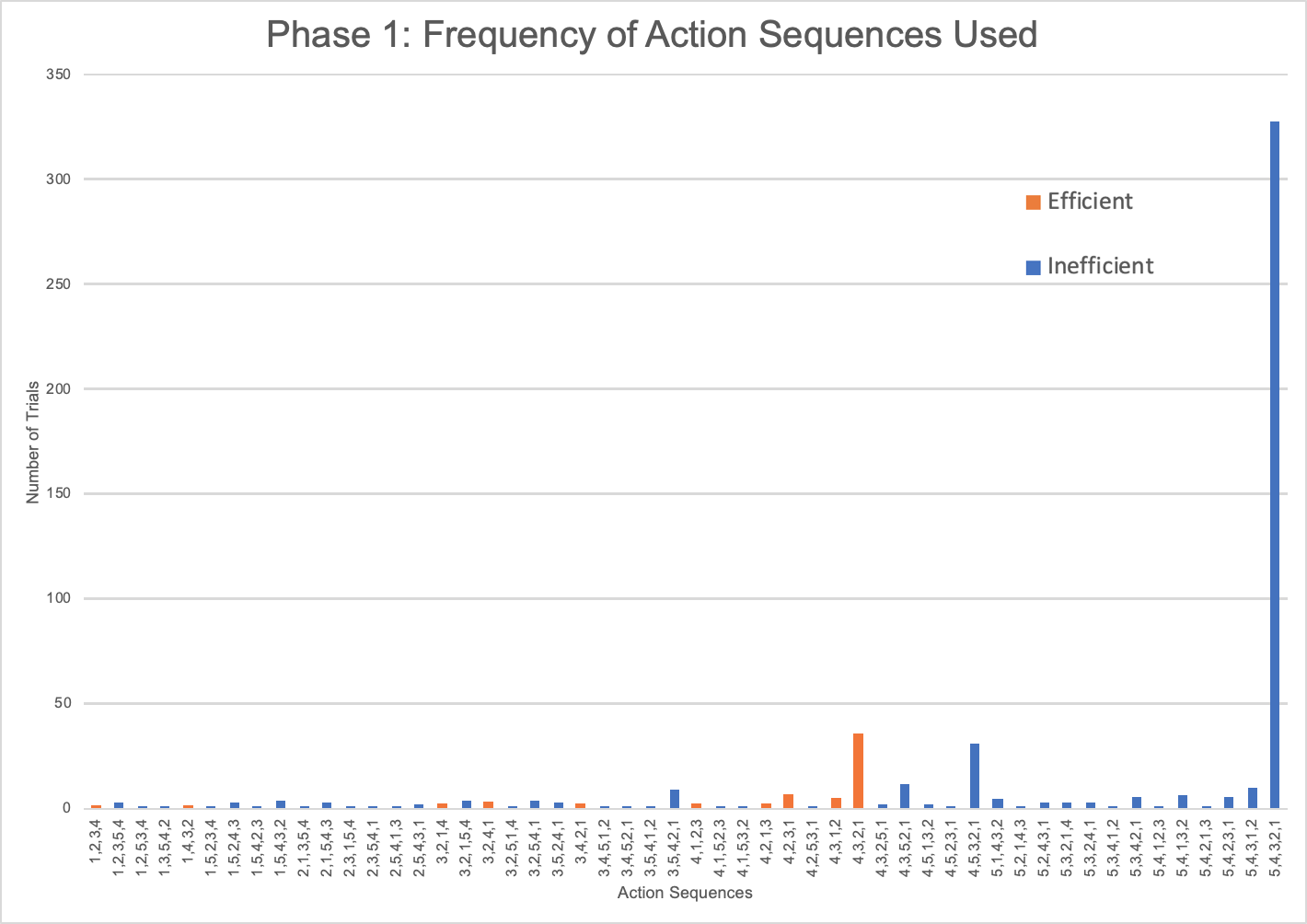

Supplement: Supplemental Information 1 [file peerj-13-19023-s001.png]
