# Supplementary material for: Fairly flexible: brown-tufted capuchins and a squirrel monkey adjust their motor responses in a foraging task
Source: PeerJ. 2025 Mar 12;13:e19023. doi: 10.7717/peerj.19023 (PMC11910149; doi:10.7717/peerj.19023)
Supplement: Supplemental Information 2 — Grey boxes indicate successive runs of the same sequence. [file peerj-13-19023-s002.pdf]

<table>
<tr><th rowspan="2">Trial</th><th>Capuchin</th><th colspan="4">Squirrel Monkeys</th></tr>
<tr><th>Nb</th><th>Ar</th><th>Co</th><th>Ec</th><th>Vi</th></tr>
<tr><td>1</td><td>5,4,3,2,1</td><td rowspan="2">5,4,3,2,1</td><td>5,3,2,4,1</td><td>5,4,3,2,1</td><td rowspan="4">5,4,3,2,1</td></tr>
<tr><td>2</td><td>4,3,5,2,1</td><td rowspan="2">5,4,3,2,1</td><td>5,4,1,3,2</td></tr>
<tr><td>3</td><td>3,4,5,2,1</td><td>5,3,4,2,1</td><td>5,3,4,1,2</td></tr>
<tr><td>4</td><td>3,4,2,1</td><td>5,1,4,3,2</td><td>3,5,4,2,1</td><td rowspan="2">4,5,3,2,1</td></tr>
<tr><td>5</td><td>3,5,4,1,2</td><td>5,2,4,3,1</td><td>5,4,2,3,1</td><td>5,4,1,3,2</td></tr>
<tr><td>6</td><td>4,2,1,3</td><td rowspan="2">5,4,3,2,1</td><td>4,5,3,2,1</td><td>1,5,2,3,4</td><td>5,4,3,1,2</td></tr>
<tr><td>7</td><td>3,4,2,1</td><td>4,3,5,2,1</td><td>1,2,3,4</td><td>3,2,5,4,1</td></tr>
<tr><td>8</td><td>4,5,2,3,1</td><td rowspan="3">4,3,2,1</td><td rowspan="2">5,4,3,2,1</td><td rowspan="3">1,2,3,5,4</td><td>5,4,3,2,1</td></tr>
<tr><td>9</td><td rowspan="2">4,2,3,1</td><td>5,1,4,3,2</td></tr>
<tr><td>10</td><td>5,4,1,2,3</td><td>3,5,4,2,1</td></tr>
<tr><td>11</td><td rowspan="2">4,5,3,2,1</td><td>5,4,3,2,1</td><td>5,4,3,2,1</td><td>1,2,5,3,4</td><td rowspan="3">5,4,3,2,1</td></tr>
<tr><td>12</td><td>1,4,3,2</td><td>2,1,3,5,4</td><td>5,4,3,1,2</td></tr>
<tr><td>13</td><td>5,3,4,2,1</td><td>5,4,3,2,1</td><td>3,5,4,2,1</td><td>1,5,2,4,3</td></tr>
<tr><td>14</td><td>4,5,3,2,1</td><td>4,5,3,2,1</td><td>5,3,2,1,4</td><td>4,5,1,3,2</td><td>4,3,5,2,1</td></tr>
<tr><td>15</td><td rowspan="2">5,4,3,2,1</td><td>5,4,3,2,1</td><td>5,4,3,2,1</td><td>1,5,2,4,3</td><td rowspan="80">5,4,3,2,1</td></tr>
<tr><td>16</td><td>5,4,2,3,1</td><td rowspan="3">5,4,2,3,1</td><td>3,4,5,1,2</td></tr>
<tr><td>17</td><td rowspan="2">4,5,3,2,1</td><td>4,5,3,2,1</td><td>3,5,4,2,1</td></tr>
<tr><td>18</td><td>5,4,3,2,1</td><td>5,4,3,2,1</td></tr>
<tr><td>19</td><td>4,3,2,1</td><td>4,3,2,5,1</td><td>3,5,2,4,1</td><td>4,3,2,1</td></tr>
<tr><td>20</td><td>4,3,5,2,1</td><td>4,3,2,1</td><td>5,4,3,2,1</td><td>5,3,4,2,1</td></tr>
<tr><td>21</td><td rowspan="4">5,4,3,2,1</td><td>5,4,3,2,1</td><td>5,4,3,1,2</td><td>5,4,3,2,1</td></tr>
<tr><td>22</td><td>4,3,5,2,1</td><td>5,4,3,2,1</td><td>5,4,3,1,2</td></tr>
<tr><td>23</td><td>4,3,2,1</td><td>1,5,4,3,2</td><td>5,4,3,2,1</td></tr>
<tr><td>24</td><td>4,5,3,2,1</td><td>4,5,1,3,2</td><td>4,1,5,2,3</td></tr>
<tr><td>25</td><td>4,5,3,2,1</td><td>5,4,3,2,1</td><td>5,4,3,1,2</td><td>1,5,4,2,3</td></tr>
<tr><td>26</td><td rowspan="5">5,4,3,2,1</td><td rowspan="3">4,3,2,1</td><td rowspan="2">3,5,4,2,1</td><td>4,5,3,2,1</td></tr>
<tr><td>27</td><td>4,3,5,2,1</td></tr>
<tr><td>28</td><td rowspan="9">5,4,3,2,1</td><td>4,3,2,5,1</td></tr>
<tr><td>29</td><td rowspan="2">5,4,3,2,1</td><td rowspan="5">5,4,3,2,1</td></tr>
<tr><td>30</td></tr>
<tr><td>31</td><td rowspan="3">4,3,2,1</td><td>4,5,3,2,1</td></tr>
<tr><td>32</td><td rowspan="5">5,4,3,2,1</td></tr>
<tr><td>33</td></tr>
<tr><td>34</td><td rowspan="8">5,4,3,2,1</td><td>5,4,3,1,2</td></tr>
<tr><td>35</td><td rowspan="4">5,4,3,2,1</td></tr>
<tr><td>36</td></tr>
<tr><td>37</td><td>4,3,2,1</td><td rowspan="2">4,5,3,2,1</td></tr>
<tr><td>38</td><td rowspan="13">5,4,3,2,1</td></tr>
<tr><td>39</td><td rowspan="4">5,4,3,2,1</td><td>4,3,2,1</td></tr>
<tr><td>40</td><td rowspan="9">5,4,3,2,1</td></tr>
<tr><td>41</td></tr>
<tr><td>42</td><td>4,5,3,2,1</td></tr>
<tr><td>43</td><td rowspan="7">5,4,3,2,1</td><td>5,4,2,3,1</td></tr>
<tr><td>44</td><td rowspan="2">5,4,3,2,1</td></tr>
<tr><td>45</td></tr>
<tr><td>46</td><td>5,4,1,3,2</td></tr>
<tr><td>47</td><td>4,5,3,2,1</td></tr>
<tr><td>48</td><td rowspan="3">5,4,3,2,1</td></tr>
<tr><td>49</td><td rowspan="4">4,3,2,1</td></tr>
<tr><td>50</td><td>4,3,2,1</td></tr>
<tr><td>51</td><td rowspan="2">5,4,3,2,1</td><td>4,3,5,2,1</td><td>3,5,4,2,1</td></tr>
<tr><td>52</td><td rowspan="3">5,4,3,2,1</td><td>5,4,3,2,1</td></tr>
<tr><td>53</td><td>4,3,5,2,1</td><td>4,5,3,2,1</td><td>4,2,5,3,1</td></tr>
<tr><td>54</td><td rowspan="10">5,4,3,2,1</td><td>3,5,4,2,1</td><td>4,3,1,2</td></tr>
<tr><td>55</td><td>5,1,4,3,2</td><td>4,3,5,2,1</td><td>4,2,3,1</td></tr>
<tr><td>56</td><td>5,4,3,2,1</td><td rowspan="7">5,4,3,2,1</td><td rowspan="2">5,4,3,2,1</td></tr>
<tr><td>57</td><td>4,5,3,2,1</td></tr>
<tr><td>58</td><td rowspan="7">5,4,3,2,1</td><td>3,2,1,5,4</td></tr>
<tr><td>59</td><td rowspan="2">4,3,2,1</td></tr>
<tr><td>60</td></tr>
<tr><td>61</td><td rowspan="5">5,4,3,2,1</td></tr>
<tr><td>62</td></tr>
<tr><td>63</td><td>1,5,4,3,2</td></tr>
<tr><td>64</td><td>4,3,2,1</td><td>5,4,3,2,1</td></tr>
<tr><td>65</td><td>4,5,3,2,1</td><td>3,2,5,4,1</td><td>4,5,3,2,1</td></tr>
<tr><td>66</td><td rowspan="4">5,4,3,2,1</td><td>4,3,2,1</td><td>2,5,4,1,3</td><td>3,5,2,4,1</td></tr>
<tr><td>67</td><td rowspan="2">5,4,3,2,1</td><td>5,4,3,2,1</td><td>3,2,4,1</td></tr>
<tr><td>68</td><td>3,2,5,1,4</td><td>5,2,1,4,3</td></tr>
<tr><td>69</td><td>4,2,3,1</td><td rowspan="2">5,4,3,2,1</td><td>5,4,3,2,1</td></tr>
<tr><td>70</td><td>4,3,5,2,1</td><td>5,4,3,2,1</td><td>4,3,2,1</td></tr>
<tr><td>71</td><td rowspan="3">5,4,3,2,1</td><td>4,3,2,1</td><td>4,5,3,2,1</td><td rowspan="8">5,4,3,2,1</td></tr>
<tr><td>72</td><td>4,5,3,2,1</td><td>5,4,3,2,1</td></tr>
<tr><td>73</td><td rowspan="3">5,4,3,2,1</td><td>3,5,4,2,1</td></tr>
<tr><td>74</td><td>4,3,5,2,1</td><td rowspan="4">5,4,3,2,1</td></tr>
<tr><td>75</td><td rowspan="5">5,4,3,2,1</td></tr>
<tr><td>76</td><td>5,1,4,3,2</td></tr>
<tr><td>77</td><td>5,4,3,2,1</td></tr>
<tr><td>78</td><td>5,4,3,1,2</td><td>4,3,2,1</td></tr>
<tr><td>79</td><td rowspan="2">4,3,1,2</td><td rowspan="3">5,4,3,2,1</td><td>5,3,2,1,4</td></tr>
<tr><td>80</td><td>4,5,3,2,1</td><td rowspan="2">5,4,3,2,1</td></tr>
<tr><td>81</td><td rowspan="3">5,4,3,2,1</td><td rowspan="2">4,3,2,1</td></tr>
<tr><td>82</td><td>1,5,4,3,2</td><td>5,1,4,3,2</td></tr>
<tr><td>83</td><td>5,4,3,2,1</td><td>5,4,3,2,1</td><td rowspan="3">5,4,3,2,1</td></tr>
<tr><td>84</td><td>4,5,3,2,1</td><td>3,2,1,4</td><td>4,1,2,3</td></tr>
<tr><td>85</td><td rowspan="4">5,4,3,2,1</td><td>5,4,3,2,1</td><td>5,3,4,2,1</td></tr>
<tr><td>86</td><td>5,3,2,4,1</td><td>4,2,3,1</td><td>1,5,2,4,3</td></tr>
<tr><td>87</td><td>5,4,2,1,3</td><td>2,3,5,4,1</td><td rowspan="4">5,4,3,2,1</td></tr>
<tr><td>88</td><td>5,3,4,2,1</td><td>5,4,3,2,1</td></tr>
<tr><td>89</td><td>4,5,3,2,1</td><td>4,3,1,2</td><td>2,1,5,4,3</td></tr>
<tr><td>90</td><td rowspan="4">5,4,3,2,1</td><td>5,4,3,2,1</td><td rowspan="2">5,4,3,2,1</td></tr>
<tr><td>91</td><td>5,4,3,1,2</td><td>5,4,3,1,2</td></tr>
<tr><td>92</td><td>4,1,2,3</td><td>5,4,1,3,2</td><td rowspan="7">5,4,3,2,1</td></tr>
<tr><td>93</td><td>5,4,3,2,1</td><td>4,1,5,3,2</td></tr>
<tr><td>94</td><td rowspan="2">4,5,3,2,1</td><td>5,3,2,1,4</td><td>4,3,2,1</td></tr>
<tr><td>95</td><td>4,3,2,1</td><td>1,5,4,3,2</td><td></td></tr>
<tr><td>96</td><td rowspan="2">5,4,3,2,1</td><td>4,2,1,3</td><td rowspan="2">4,2,3,1</td><td></td></tr>
<tr><td>97</td><td>5,4,3,2,1</td><td></td></tr>
<tr><td>98</td><td rowspan="2">4,5,3,2,1</td><td>3,2,4,1</td><td>4,3,2,1</td><td></td></tr>
<tr><td>99</td><td rowspan="5">5,4,3,2,1</td><td>3,2,1,4</td><td></td><td></td></tr>
<tr><td>100</td><td></td><td>3,2,4,1</td><td></td><td></td></tr>
<tr><td>101</td><td></td><td>3,2,1,5,4</td><td></td><td></td></tr>
<tr><td>102</td><td></td><td>4,3,2,1</td><td></td><td></td></tr>
<tr><td>103</td><td></td><td>3,5,2,4,1</td><td></td><td></td></tr>
<tr><td>104</td><td></td><td>5,3,4,2,1</td><td>3,2,1,5,4</td><td></td><td></td></tr>
<tr><td>105</td><td></td><td>5,4,3,1,2</td><td>2,1,5,4,3</td><td></td><td></td></tr>
<tr><td>106</td><td></td><td rowspan="3">5,4,3,2,1</td><td>5,3,2,4,1</td><td></td><td></td></tr>
<tr><td>107</td><td></td><td>2,3,1,5,4</td><td></td><td></td></tr>
<tr><td>108</td><td></td><td>5,4,3,2,1</td><td></td><td></td></tr>
<tr><td>109</td><td></td><td>4,3,2,1</td><td>5,2,4,3,1</td><td></td><td></td></tr>
<tr><td>110</td><td></td><td>5,4,1,3,2</td><td>5,4,3,2,1</td><td></td><td></td></tr>
<tr><td>111</td><td></td><td>5,4,3,2,1</td><td>4,3,5,2,1</td><td></td><td></td></tr>
<tr><td>112</td><td></td><td>4,3,2,1</td><td>2,5,4,3,1</td><td></td><td></td></tr>
<tr><td>113</td><td></td><td>5,4,3,2,1</td><td>2,1,5,4,3</td><td></td><td></td></tr>
<tr><td>114</td><td></td><td>5,4,1,3,2</td><td rowspan="2">5,4,3,2,1</td><td></td><td></td></tr>
<tr><td>115</td><td></td><td>4,3,2,1</td><td></td><td></td></tr>
<tr><td>116</td><td></td><td rowspan="5">5,4,3,2,1</td><td rowspan="2">3,2,5,4,1</td><td></td><td></td></tr>
<tr><td>117</td><td></td><td></td><td></td></tr>
<tr><td>118</td><td></td><td>5,4,1,3,2</td><td></td><td></td></tr>
<tr><td>119</td><td></td><td>5,2,4,3,1</td><td></td><td></td></tr>
<tr><td>120</td><td></td><td>2,5,4,3,1</td><td></td><td></td></tr>
<tr><td>121</td><td></td><td>4,3,1,2</td><td>3,2,1,5,4</td><td></td><td></td></tr>
<tr><td>122</td><td></td><td></td><td>1,3,5,4,2</td><td></td><td></td></tr>
</table>
