# Supplementary material for: Fairly flexible: brown-tufted capuchins and a squirrel monkey adjust their motor responses in a foraging task
Source: PeerJ. 2025 Mar 12;13:e19023. doi: 10.7717/peerj.19023 (PMC11910149; doi:10.7717/peerj.19023)
Supplement: Supplemental Information 3 — Grey boxes indicate successive runs of the same sequence. [file peerj-13-19023-s003.pdf]

<table>
<tr><th>Trial</th><th>Dv</th><th>Mt</th></tr>
<tr><td>1</td><td rowspan="3">5,3,2,1</td><td>3,2,1</td></tr>
<tr><td>2</td><td>2,3,1</td></tr>
<tr><td>3</td><td>2,1</td></tr>
<tr><td>4</td><td>4,2,1</td><td>5,4,3,2,1</td></tr>
<tr><td>5</td><td>4,3,2,1</td><td rowspan="2">2,3,1</td></tr>
<tr><td>6</td><td>3,2,1</td></tr>
<tr><td>7</td><td>4,2,1</td><td>3,2,1</td></tr>
<tr><td>8</td><td>3,2,1</td><td>2,1</td></tr>
<tr><td>9</td><td>5,3,2,1</td><td>3,2,1</td></tr>
<tr><td>10</td><td>4,2,1</td><td>2,1</td></tr>
<tr><td>11</td><td>3,2,1</td><td>4,3,2,1</td></tr>
<tr><td>12</td><td>4,3,2,1</td><td>3,2,4,1</td></tr>
<tr><td>13</td><td>3,2,1</td><td>2,1</td></tr>
<tr><td>14</td><td>4,3,2,1</td><td>2,3,1</td></tr>
<tr><td>15</td><td>3,2,1</td><td rowspan="2">3,2,1</td></tr>
<tr><td>16</td><td>2,1</td></tr>
<tr><td>17</td><td>3,2,1</td><td>2,1</td></tr>
<tr><td>18</td><td>2,1</td><td>2,3,1</td></tr>
<tr><td>19</td><td>5,3,2,1</td><td rowspan="2">2,1</td></tr>
<tr><td>20</td><td rowspan="3">4,3,2,1</td></tr>
<tr><td>21</td><td>4,5,3,2,1</td></tr>
<tr><td>22</td><td>2,3,1</td></tr>
<tr><td>23</td><td rowspan="2">2,1</td><td>3,2,1</td></tr>
<tr><td>24</td><td rowspan="3">2,1</td></tr>
<tr><td>25</td><td>5,3,2,1</td></tr>
<tr><td>26</td><td>3,2,1</td></tr>
<tr><td>27</td><td>5,4,3,2,1</td><td>1,3,2</td></tr>
<tr><td>28</td><td>5,3,2,1</td><td rowspan="2">2,1</td></tr>
<tr><td>29</td><td>4,3,2,1</td></tr>
<tr><td>30</td><td>5,3,2,1</td><td rowspan="2">3,2,1</td></tr>
<tr><td>31</td><td>3,2,1</td></tr>
<tr><td>32</td><td>5,4,3,2,1</td><td>2,1</td></tr>
<tr><td>33</td><td>5,3,2,1</td><td>3,2,1</td></tr>
<tr><td>34</td><td>3,2,1</td><td rowspan="2">2,1</td></tr>
<tr><td>35</td><td>5,3,2,1</td></tr>
<tr><td>36</td><td>3,2,1</td><td>3,2,1</td></tr>
<tr><td>37</td><td rowspan="4">5,3,2,1</td><td rowspan="4">2,1</td></tr>
<tr><td>38</td></tr>
<tr><td>39</td></tr>
<tr><td>40</td></tr>
<tr><td>41</td><td>2,1</td><td>2,3,1</td></tr>
<tr><td>42</td><td>5,3,2,1</td><td>2,1</td></tr>
<tr><td>43</td><td>5,4,3,2,1</td><td>3,2,1</td></tr>
<tr><td>44</td><td>4,2,1</td><td>2,1</td></tr>
<tr><td>45</td><td>4,3,2,1</td><td>3,2,1</td></tr>
<tr><td>46</td><td rowspan="2">5,4,3,2,1</td><td rowspan="4">2,1</td></tr>
<tr><td>47</td></tr>
<tr><td>48</td><td>4,2,1</td></tr>
<tr><td>49</td><td rowspan="2">4,3,2,1</td></tr>
<tr><td>50</td><td>3,2,1</td></tr>
</table>
